# Fungal β-Glucans Enhance Lactic Acid Bacteria Growth by Shortening Their Lag Phase and Increasing Growth Rate

**DOI:** 10.3390/microorganisms13061313

**Published:** 2025-06-05

**Authors:** Andrea Bukša, Filip Petrović, Željka Maglica

**Affiliations:** Faculty of Biotechnology and Drug Development, University of Rijeka, Radmile Matejčić 2, 51000 Rijeka, Croatia; andrea.buksa@uniri.hr (A.B.); petrovicf@outlook.com (F.P.)

**Keywords:** prebiotics, fungal polysaccharides, β-glucans, probiotics, *Lactobacillus*, *Lacticaseibacillus*, synbiotics

## Abstract

The gut microbiome has a significant role in general health and well-being. Novel types of prebiotics, such as fungal polysaccharides, show potential for the formulation of new synbiotic formulations. However, little is known about the underlying mechanisms of the prebiotic effects of such compounds. This study investigated the prebiotic properties of fungal glucan extracts from *Pleurotus ostreatus*, *Lentinula edodes*, and *Saccharomyces cerevisiae*, employing a novel high-throughput method based on optical density measurements. This approach enabled the simultaneous screening of the effects of multiple extracts on six different strains of probiotic bacteria. Experiments were conducted to evaluate the effect of the extracts on the growth dynamics (the duration of the lag phase and the growth rate) of probiotic strains of the genera *Lactobacillus* and *Lacticaseibacillus* and on pathogenic bacteria. Fungal polysaccharide supplementation, particularly with their β-glucans, significantly shortened the lag phase by an average of 7–8 h in all tested strains and increased the growth rate by 2-fold in four strains of lactic acid bacteria. Different magnitudes of effects were observed across the various strain–extract combinations. This study lays the groundwork for elucidating the mechanism by which fungal β-glucans stimulate growth in probiotic bacteria and for the rapid screening of optimal combinations for formulating innovative synbiotics.

## 1. Introduction

Recent advances in microbiome research have emphasized the crucial role of probiotic microorganisms in maintaining intestinal health and modulating systemic physiological functions in the human body [1]. Probiotics are defined as “live microorganisms that, when administered in adequate amounts, confer a health benefit on the host” (FAO/WHO 2001). These beneficial microbes contribute to gut homeostasis through various mechanisms, including competitive exclusion of pathogens, immunomodulation, and enhancement of the intestinal epithelial barrier [2]. Among the most studied probiotics are lactic acid bacteria, particularly species belonging to the genera *Lactobacillus*, *Lacticaseibacillus*, *Lactococcus*, and *Bifidobacterium*. These Gram-positive, non-spore-forming, rod- or coccus-shaped bacteria are predominantly facultative anaerobes or microaerophiles, and they typically employ homofermentative metabolism, converting carbohydrates such as glucose and lactose into lactic acid [3]. In this study, we focused on lactic acid bacteria belonging to the genera *Lactobacillus* and *Lacticaseibacillus*, which include commercially significant strains such as *L. casei*, *L. rhamnosus GG*, *L. plantarum*, and *L. johnsonii*. These species are frequently used in functional foods and probiotic supplements due to their well-documented health-promoting properties.

Prebiotics are non-digestible food ingredients that selectively stimulate the growth or activity of beneficial microorganisms in the host’s gastrointestinal tract. These compounds are typically dietary fibers, which are resistant to digestion in the upper gastrointestinal tract but are readily fermented by gut microbiota. Among the most extensively studied and widely used prebiotics are galactooligosaccharides (GOS) and fructooligosaccharides (FOS) [4]. Other well-established prebiotics include inulin, lactulose, and polydextrose. Recently, novel sources of bioactive carbohydrates have been identified in various plant and fungal species, expanding the range of compounds with prebiotic potential. The primary microbial targets of prebiotics are members of the genera *Lactobacillus* and *Bifidobacterium*, which are associated with gut health. A positive prebiotic effect is typically reflected by an increased abundance of these beneficial bacteria, often accompanied by suppression of opportunistic or pathogenic species through competitive exclusion and metabolic by-products.

Some of the most extensively studied edible and medicinal mushrooms belong to the genera *Pleurotus*, *Lentinula*, and *Ganoderma*, which have been valued for centuries for both their nutritional and therapeutic properties. In this study, we focused on oyster mushrooms (*Pleurotus ostreatus*) and shiitake mushrooms (*Lentinula edodes*). Oyster mushrooms have been reported to exhibit immunostimulatory activity [5], while shiitake mushrooms are recognized as rich sources of vitamins, proteins, lipids, and minerals [6]. Fungal biomass is predominantly composed of polysaccharides, which can constitute over 75% of the total dry mass in many species [7]. In *L. edodes*, the polysaccharide content has been reported at approximately 2 mg/g of dry matter, whereas in *Pleurotus* species, concentrations can range from 2.2 to 5.3 mg/g [8]. These mushroom-derived polysaccharides are primarily glucose polymers (glucans), which may be linear or branched and contain various glycosidic linkages. The most prevalent types include β-1,3- and β-1,6-glucans as well as α-1,3-glucans. Among these, β-1,3-glucans are the most abundant and are widely recognized for their immunomodulatory effects in humans [9].

Beyond their immune-stimulating properties, β-glucans have also gained attention as potential prebiotic compounds, based on studies showing their ability to enhance the growth of beneficial gut microbes, particularly lactobacilli and bifidobacteria. In particular, oligomeric fragments of β-glucans have demonstrated strong bioactivity and are being explored as novel functional prebiotic agents [9]. A study by Synytsya et al. demonstrated that polysaccharide extracts derived from *Pleurotus ostreatus* and *Pleurotus eryngii* can stimulate the growth of several probiotic bacterial strains belonging to the genera *Lactobacillus*, *Bifidobacterium*, and *Enterococcus*. Both mushroom extracts exhibited notable growth-promoting effects on *Lactobacillus* spp., although the magnitude of the response varied among strains. In contrast, the stimulatory effect on bifidobacteria was comparatively modest, suggesting a greater selectivity toward lactobacilli within the probiotic group tested [10]. Other studies have shown that certain probiotic microorganisms are capable of utilizing β-glucans as a carbon source, which can positively influence their growth dynamics and enhance lactic acid production in the gastrointestinal tract [11,12]. Additionally, certain β-glucans of bacterial origin have been shown to enhance the adhesion capacity of *Lactobacillus* strains to intestinal epithelial cells, thereby facilitating their colonization and proliferation within the host gastrointestinal tract [13].

It is still unclear, however, what the mechanism is behind the prebiotic effects of these polysaccharides. The aim of this study was to investigate whether fungal glucans derived from the mushrooms *Pleurotus ostreatus* and *Lentinula edodes* could serve as a novel class of prebiotic compounds for the development of enhanced synbiotic formulations. We hypothesized that fungal polysaccharides, especially β-glucans, would possess strain-specific prebiotic effects on probiotic bacteria from the genera *Lactobacillus* and *Lacticaseibacillus*, promoting their growth and potentially eliciting additional beneficial responses. An important question guiding this research was whether these polysaccharides act solely as carbon sources or whether they exert their effects through additional mechanisms, such as modulating growth kinetics of those bacteria. Specifically, we sought to identify optimal combinations of probiotic strains and fungal polysaccharide sources and to evaluate their effects on key microbial growth parameters.

## 2. Materials and Methods

### 2.1. Extraction of Polysaccharides from Pleurotus ostreatus and Lentinula edodes

Fresh *Pleurotus ostreatus* and *Lentinula edodes* mushrooms were locally sourced from Rijeka, Croatia. Samples were dehydrated in a food dehydrator, ground using a coffee grinder, and sifted through a <1 mm mesh. Hot water extraction was performed as follows. A total of 30 g of mushroom powder was mixed with 500 mL of ultrapure water (Thermo Scientific GenPure XCAD Plus) in a glass Erlenmeyer flask and boiled for 120 min [14]. After cooling to room temperature, the extract was first filtered through gauze and then through 1 µm pore filter paper using a Büchner funnel under vacuum. The filtrates were dried at 100 °C (IN55, Memmert) until complete evaporation [15]. A 30% (*w*/*w*) stock solution of each dried extract was prepared in ultrapure water with vigorous shaking. Total glucan content of extracts was determined using the Megazyme β-Glucan Assay Kit (Yeast and Mushroom) (Megazyme, Bray, Co., Wicklow, Ireland), following the manufacturer’s protocol [16]. The dried *Pleurotus ostreatus* fruiting bodies contained an average of 32.7% total glucans (*w*/*w*) in dry matter, while *Lentinula edodes* contained an average of 20.0%, with more than 90% of these being β-glucans. These values are consistent with previously reported data in the literature [8]. We analyzed the *Pleurotus ostreatus* extract further by using a Malvern Zetasizer instrument. Each sample was measured ten times, and the mean particle diameter was calculated. The *Pleurotus ostreatus* water extract had the average diameter of 0.2 µm, while the commercial β-glucan extract from *Pleurotus ostreatus* (Yohncan, China) showed a slightly larger mean diameter of 0.3 µm. Since the prebiotic stimulation effect obtained using above mentioned *Pleurotus ostreatus* extract was consistent with the results obtained using a characterized, commercial, *Pleurotus ostreatus* 30% β-D-glucan extract (Yohncan, China), no further chemical characterization of the in-house extracts was performed.

### 2.2. Saccharomyces cerevisiae Extract Solution

Commercial yeast glucans, i.e., Goldcell *Saccharomyces cerevisiae* β-glucan extract (Biorigin, Brazil), kindly provided by the pharmaceutical company Yasenka Ltd., Croatia, were used for initial experiments to evaluate the effects of yeast extracts on *Lactobacillus* and *Lacticaseibacillus* strains. The manufacturers state that the β-glucan content is 75% (*w*/*w* in dry matter).

In addition, a commercial standardized yeast β-glucan was obtained as a standard in the Megazyme β-glucans Assay Kit (Megazyme, Bray, Co. Wicklow, Ireland), and it is claimed that is contains 49% β-glucans. We prepared 0.5% (*w*/*w*) of this initial standard preparation but observed high turbidity. Therefore, we left the insoluble particles to precipitate overnight at 4 C. Both the initial standard preparation and the supernatant were then tested to evaluate their prebiotic potential.

Bacto™ yeast extract (Thermo Fisher Scientific, Waltham, MA, USA) that contains autolyzed water-soluble yeast cells was also tested for its prebiotic potential.

### 2.3. Bacterial Strains Used

In this study, we used representative strains from both the *Lactobacillus* and *Lacticaseibacillus* genera. The *Lactobacillus* group included *L. acidophilus* 145 and *L. helveticus* (ATCC 15009), while the *Lacticaseibacillus* group comprised *L. casei* (ATCC 393), *L. rhamnosus* GG (ATCC 53103), *L. paracasei* (ATCC 334), and *L. paracasei* subsp. *paracasei* CCM 1753. As representatives of pathogenic and opportunistic strains, we used *Escherichia coli* DH5α (prepared as competent using the TSS method and referenced in the manuscript as strain 2) and *E. coli* ATCC 11229 (referenced in the manuscript as strain 1)*, Listeria monocytogenes* hemolytic EGD strain (serovar1/2a)*,* and the clinical isolate of *Salmonella enterica* serotype Typhimurium 3064.

### 2.4. Bacterial Cultivation and Growth Kinetics Monitoring

Liquid cultures of *Lactobacillus* and *Lacticaseibacillus* strains were grown at 37 °C in MRS broth (Carl Roth GmbH + Co., KG, Karlsruhe, Germany), while solid cultures were maintained on MRS agar (Biolife, Italy). For pathogenic strains (*E. coli*, *Salmonella enterica* serovar Typhimurium, and *Listeria monocytogenes*), LB broth (Carl Roth) was used as the growth medium.

Bacterial growth was tracked using a HIDEX Sense microplate reader with the default software supplied with the instrument, at 37 °C for 24 h, with absorbance measurements recorded every hour. This high-throughput approach was based on a method previously described by Watson et al. [17]. All growth assays were conducted in 96-well flat-bottom microplates with plastic lids (Thermo Fisher Scientific). Each well was filled with 180 µL of sterile MRS broth (Carl Roth GmbH & Co. KG, Germany). For test conditions, 18 µL of polysaccharide extract was added to designated sample wells, while saline solution was added to non-supplemented control wells (control). Blank (sterility control) wells received polysaccharide extract and saline solution but no bacterial inoculum. Sample and control wells were inoculated with 18 µL of overnight bacterial culture diluted to 10^−4^, while blank wells received 18 µL of saline solution instead of bacteria. To minimize evaporation effects caused by warm air circulation during incubation, the peripheral wells of the plate were filled with sterile water. To create microaerophilic conditions, the outermost wells were inoculated with 18 µL of a 10^−1^ dilution of overnight culture to serve as CO_2_ producers, and then, the plate was sealed with parafilm. This setup improved measurement accuracy by promoting even bacterial growth throughout the liquid medium and preventing cell aggregation at the bottom, which often occurs under fully aerobic conditions. However, this method does not offer full anaerobic conditions, and it is not suitable for incubations longer than 24 h. Six replicates of each strain/treatment combination were tested. Growth characteristics (lag time and growth rate) were analyzed according to the model of Baranyi and Roberts, using DMFit 3.5 software [18].

### 2.5. Statistical Analysis

The statistical significance was analyzed with the STATISTICA v.14 program package, using two-sample Student’s *t*-test. All results are presented as mean value ± standard deviation (SD). Differences were considered statistically significant when *p* < 0.05.

## 3. Results

### 3.1. Effect of Pleurotus ostreatus and Lentinula edodes Glucan Extracts on the Growth of Lacticaseibacillus casei

**Figure 1:**
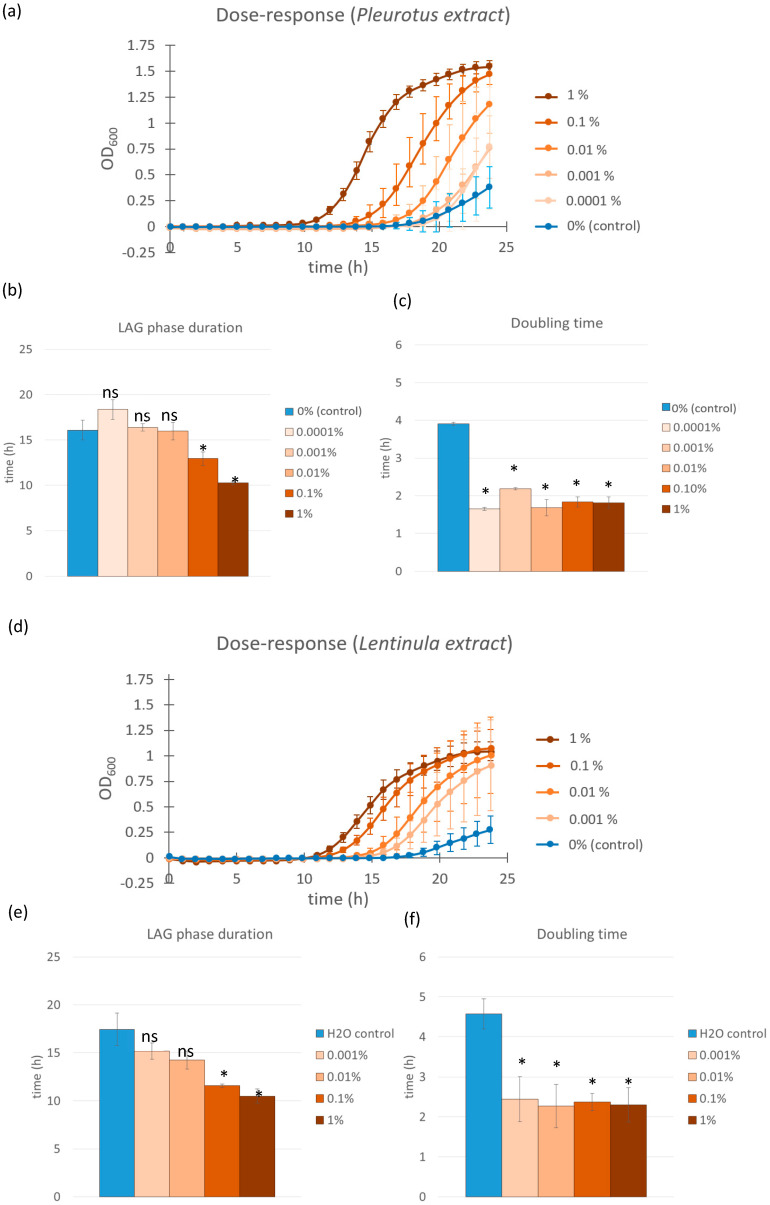
Growth curves of *L. casei* supplemented with different concentrations of *Pleurotus ostreatus* and *Lentinula edodes* extracts (**a**,**d**). Control cultures were supplemented with equal volumes of saline solution (blue line). Error bars represent standard errors of the means from six readings for each time point. Lag phase duration (**b**) and doubling time (**c**) were calculated from the growth curves above for cultures supplemented with *Pleurotus ostreatus* extract solution and those supplemented with *Lentinula edodes* extract solutions (**e**,**f**). Color coding is the same as in the growth curves above. Statistical significance was assessed using Student’s *t*-test. * *p* < 0.0001 compared to the control group; “ns” for non-significant.

To investigate the effect of *Pleurotus ostreatus* and *Lentinula edodes* glucan extracts on the probiotic bacterium *L. casei*, we monitored bacterial growth over 24 h. The most rapid onset of exponential growth—defined as the shortest lag phase duration—was observed in samples treated with 1.0% *P. ostreatus* extract, which entered the exponential phase at approximately t = 10.3 h and exhibited a doubling time of 1.8 h (Figure 1a–c). A dose-dependent response was evident: the 0.1% extract-treated samples entered exponential growth at t = 12.9 h, followed by the 0.01% (t = 16.0 h), 0.001% (t = 16.4 h), and 0.0001% (t = 18.4 h) treatments. Despite these differences in lag phase duration, the doubling times across all extract-treated samples remained relatively similar, ranging from 1.7 to 2.2 h, and were significantly shorter (*p* < 0.0001 in all cases) than the control (t = 3.9 h). Due to the gradual sample evaporation in the plate reader during prolonged incubation, it was not possible to reliably monitor the complete growth kinetics of all samples over a longer period. However, colony-forming unit (CFU) counts performed at the stationary phase indicated no significant differences in the maximum CFU between extract-treated and control samples. Additionally, no differences were observed in the final pH of the growth medium or in auto-aggregation behavior between treated and untreated samples upon reaching the stationary phase.

A similar trend was observed for the *L. edodes* glucan extracts when used on *L. casei*. The 1.0% extract induced the earliest exponential growth onset (t = 10 h) and the greatest reduction in lag phase duration. The 0.1%, 0.01%, and 0.001% extract dilutions resulted in progressively longer lag phases (t = 11 h, t = 14 h, and t = 15 h, respectively), while the control sample entered exponential growth at t = 17 h (Figure 1d–f). These results confirm a dose-responsive effect for both mushroom extracts, with *L. edodes* and *P. ostreatus* exhibiting comparable growth-promoting activity on *L. casei*. The doubling time reduction effect was also evident in the *Lentinula edodes* extract-treated samples. All concentrations tested showed a significant decrease in doubling time (*p* < 0.001 in all cases), with values ranging from 2.3 to 2.5 h compared to the control group, which exhibited a doubling time of 4.5 h. These findings confirm that *L. edodes* extracts, similar to *P. ostreatus*, not only accelerate entry into the exponential phase but also enhance the overall growth rate of *L. casei* during this phase. 

### 3.2. Strain-Specific Growth Responses to Pleurotus ostreatus Extract in Lactobacillus and Lacticaseibacillus Species

Building on our findings with *L. casei*, we next investigated whether the favorable growth-promoting effects of the 1.0% (*w*/*w*) *Pleurotus ostreatus* polysaccharide extract extended to other probiotic strains from the *Lactobacillus* and *Lacticaseibacillus* genera. The tested strains included *L. helveticus*, *L. rhamnosus GG*, *L. acidophilus*, *L. paracasei*, and *L. paracasei* subsp. *paracasei* in addition to *L. casei*. For all subsequent experiments, we used only the 1.0% extract concentration, as it showed the most prominent effect in initial screening.

**Figure 2:**
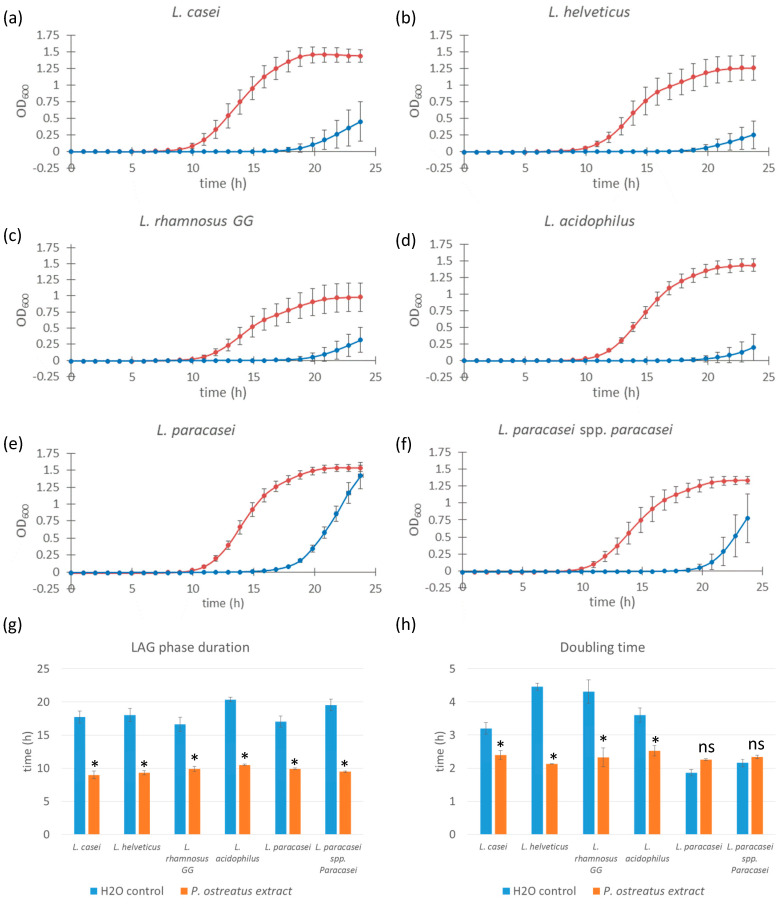
Growth curves of different *Lacticaseibacillus* and *Lactobacillus* strains supplemented with *Pleurotus ostreatus* extract. Bacterial cell cultures of *L. casei*, *L. helveticus*, *L. rhamnosus GG*, *L. acidophilus, L. paracasei,* and *L. paracasei* sp. *paracasei* (**a**–**f**) supplemented with 1.0% *w*/*w Pleurotus ostreatus* extract solution (red line) and control cultures supplemented with equal volumes of saline solution (blue line). Error bars represent standard errors of the means from six readings for each time point. Lag phase duration (**g**) and doubling time (**h**) were calculated from the growth curves above. Statistical significance was assessed using Student’s *t*-test. * *p* < 0.0001 compared to control group; “ns” for non-significant.

All six strains exhibited a significant reduction (*p*-values < 0.0001) in lag phase duration when cultured in media supplemented with the *P. ostreatus* extract compared to control samples (Figure 2). The most pronounced effect was observed in *L. acidophilus*, with the lag phase being shortened from approximately 20 h (control) to 11 h (extract-treated). A similar trend was seen in the other strains: lag phase duration decreased from 18 to 9 h in *L. casei*, 18 to 9 h in *L. helveticus*, 16 to 9 h in *L. rhamnosus GG*, 16 to 9 h in *L. paracasei*, and 18 to 9 h in *L. paracasei* subsp. *paracasei*.

Although a reduction in doubling time was observed in specific bacterial strain samples (*L. casei,* along with *L. helveticus, L. rhamnosus GG*, and *L. acidophilus*)*,* this was not true for *L. paracasei* and *L. paracasei* sp. *paracasei,* which did not show a significant decrease in doubling time compared to control samples and even demonstrated a slower growth rate in the case of *L. paracasei*. The magnitude of the reduction in doubling time varied among the strains tested. Although lag phase shortening was consistent across all strains, the effect on doubling time was more variable. A statistically significant reduction in doubling time was observed for *L. casei*, *L. helveticus*, *L. rhamnosus GG*, and *L. acidophilus*, indicating enhanced growth rates in the presence of the extract. However, in *L. paracasei* and *L. paracasei* subsp. *paracasei*, no significant decrease in doubling time was detected. In fact, *L. paracasei* showed a slight increase in doubling time, suggesting a slower growth rate relative to the control under these conditions.

These results indicate that while *P. ostreatus* polysaccharide extract consistently reduces lag phase duration across multiple strains of bacteria, its effect on growth rate (doubling time) is strain-specific, potentially reflecting differences in metabolic compatibility or extract tolerance among the *Lactobacillus* and *Lacticaseibacillus* strains.

### 3.3. Growth Effects of Commercial Yeast β-Glucan Extract on Selected Probiotic Strains

To determine whether the observed growth-promoting effects were specific to mushroom-derived polysaccharides or could be replicated using other fungal sources, we expanded our investigation to include a commercial glucan extract derived from *Saccharomyces cerevisiae*. This extract, as reported by the manufacturer, contains over 75% β-glucans (*w*/*w* in dry matter). We focused on four probiotic strains that previously demonstrated the strongest response to mushroom glucans: *L. casei*, *L. helveticus*, *L. rhamnosus GG*, and *L. paracasei*. Due to the high intrinsic optical density (OD) of the yeast β-glucan solution—which could interfere with spectrophotometric growth measurements—the extract was diluted to a final concentration of 0.025% (*w*/*w*). This issue with high OD was unique to this extract and was not observed in the previously analyzed mushroom glucan extracts.

**Figure 3:**
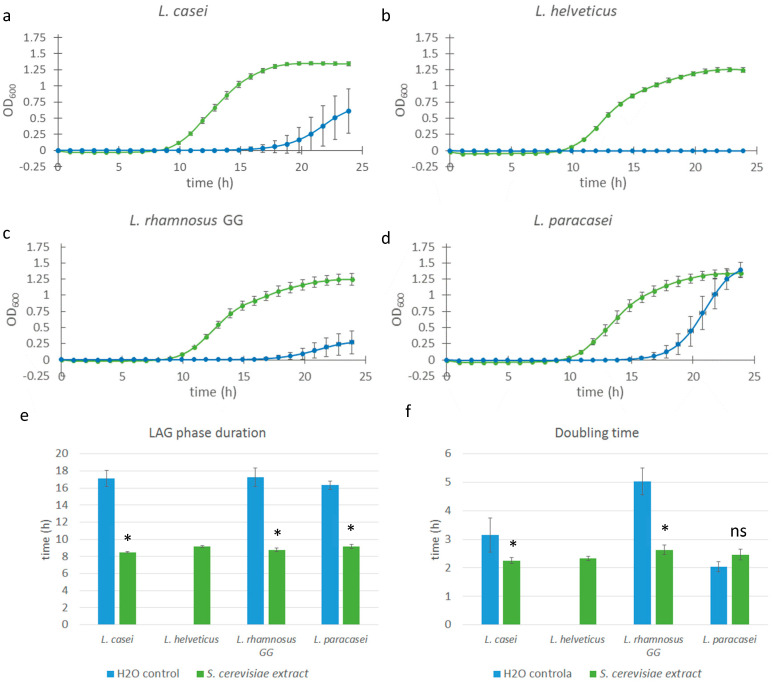
Growth curves of various *Lacticaseibacillus* and *Lactobacillus* strains supplemented with *Saccharomyces cerevisiae* extract. Bacterial cell cultures of *L. casei*, *L. helveticus*, *L. rhamnosus GG*, and *L. paracasei* (**a**–**d**) were supplemented with 0.025% *w*/*w Saccharomyces* extract solution (green line), and control cultures were supplemented with equal volumes of saline solution (blue line). Error bars represent standard errors of the means from six readings for each time point. Lag phase duration (**e**) and doubling time (**f**) were calculated from the growth curves above. Statistical significance was assessed using Student’s *t*-test. * *p* < 0.0001 compared to the control group; “ns” is non-significant.

All of the strains tested exhibited comparable responses to the yeast-derived glucan extract, with a significant reduction in lag phase duration (*p* < 0.0001 in all cases) from approximately 16–17 h (control) to 9 h in extract-treated samples—an average reduction of 7–8 h (Figure 3). This effect was consistent with the results obtained using *Pleurotus ostreatus* and *Lentinula edodes* extracts.

The effect on doubling time also mirrored previous findings: significant (*p* < 0.01) reductions were observed in *L. casei* and *L. rhamnosus GG* samples, whereas *L. paracasei* did not exhibit a notable change in growth rate compared to the control. For *L. helveticus*, assessment of extract-specific effects was limited, as no growth was observed in the control samples, probably because not enough time passed to initiate exponential growth. Nevertheless, the growth kinetics of the extract-treated *L. helveticus* cultures—including lag phase and doubling time—were comparable to those of other extract-treated strains, suggesting a similar stimulatory response.

### 3.4. Comparison of Different Yeast-Derived Preparation: Assessing the Role of β-Glucans

The yeast glucans tested so far contained 75% of β-glucans, which may mean that the effects observed could be attributed specifically to the β-glucan fraction of fungal polysaccharides. We therefore evaluated the response of *L. casei* to three different preparations derived from *Saccharomyces cerevisiae*. Two of the preparations were based on a commercial β-glucan preparation containing 49% (*w*/*w*) β-glucans, which is used as a standard for β-glucan content determination within the Megazyme kit (Megazyme, Bray, Co., Wicklow, Ireland). We observed that this preparation was prone to precipitation when dissolved in the saline solution, as previously noted for yeast β-glucans [19]. Since we did not know which fraction, the soluble or the insoluble, might have prebiotic activity, we tested 0.5% (*w*/*w*) of the initial preparation and the supernatant of this preparation, obtained after overnight precipitation at 4 °C. The third treatment consisted of a 1.0% (*w*/*w*) solution of Bacto yeast extract, which represents the concentrate of the water-soluble portion of autolyzed *Saccharomyces cerevisiae* cells. In addition to all other cellular components, this extract also contains β-glucans; however, they are in their natural form—complexed with proteins.

**Figure 4:**
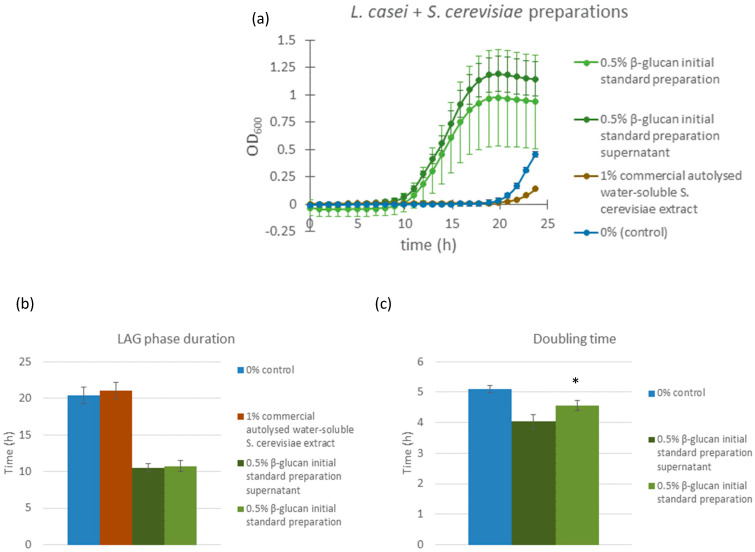
Growth curves of *L. casei* supplemented with different *Saccharomyces cerevisiae* extracts (**a**). Dark green line: bacterial cultures supplemented with 0.5% *w*/*w Saccharomyces cerevisiae* β-glucan initial standard preparation; green line: bacterial cultures supplemented with supernatant of β-glucan initial standard preparation *Saccharomyces cerevisiae* solution; brown line: bacterial cultures supplemented with 1.0% *w*/*w Saccharomyces cerevisiae* autolyzed water-soluble extract (Bacto yeast extract); blue line: control bacterial cultures, supplemented with saline solution. Error bars represent standard errors of the means from six readings for each time point. Lag phase duration (**b**) and doubling time (**c**) were calculated from the growth curves above. Statistical significance was assessed using Student’s *t*-test. * *p* < 0.0001 compared to control group; and “ns” is non-significant.

Both the β-glucan initial standard preparation and its supernatant significantly (*p* < 0.0001) shortened the lag phase of *L. casei* cultures compared to the control, with lag phase durations of 10.76 h (initial standard preparation) and 10.53 h (supernatant) versus 20.44 h in the control and 21.05 h in the Bacto yeast extract samples. Similarly, doubling times were improved in β-glucan-treated samples and measured at 4.56 h (initial standard preparation) and 4.03 h (supernatant) compared to 5.11 h in the control and 14.25 h in the Bacto yeast extract treatment. Notably, the inhomogeneity of the 49% β-glucan initial standard preparation, derived from large amount of insoluble components, resulted in higher variability (standard deviation) in the corresponding sample set, while the supernatant yielded more consistent growth responses. In contrast, samples treated with autolyzed yeast extract (Bacto) not only failed to reduce the lag phase but even showed a slight increase in lag duration compared to the control (22 h vs. 20 h) and a substantial prolongation of doubling time (Figure 4). These findings suggest that the highly purified and concentrated β-glucan component of fungal polysaccharide extracts plays a major role in modulating growth kinetics in *L. casei*.

### 3.5. Assessing the Specificity of Fungal Extract Effects in Opportunistic and Pathogenic Bacteria

To determine whether the observed growth-promoting effects of fungal β-glucans were specific to lactic acid bacteria, we examined the response of selected opportunistic and pathogenic bacterial species upon treatment with *Pleurotus ostreatus* glucan extract. The organisms tested included two strains of *Escherichia coli*, *Salmonella enterica* serovar Typhimurium, and *Listeria monocytogenes*.

**Figure 5:**
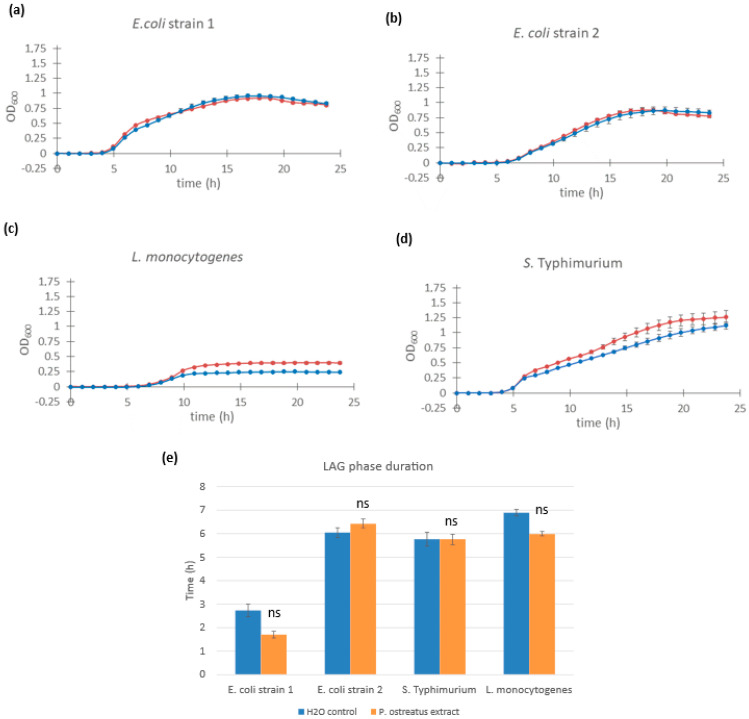
Growth curves of different pathogenic *bacteria* supplemented with *Pleurotus ostreatus* glucan extract. Cultures of *Escherichia coli* strain 1 (**a**), *Escherichia coli* strain 2 (**b**), *Listeria monocytogenes* (**c**), and *Salmonella enterica* serovar Typhimurium (**d**) supplemented with 1.0% *w*/*w Pleurotus ostreatus* glucan extract (red line) and control cultures supplemented with equal volumes of saline solution (blue line). Error bars represent standard errors of the means from six readings for each time point. The lag phase duration (**e**) was calculated for *E. coli*, *Listeria monocytogenes*, and *Salmonella enterica* serovar Typhimurium from the growth curves above. Statistical significance was assessed using Student’s *t*-test. * *p* < 0.0001 compared to control group; “ns” is non-significant.

Neither of the *E. coli* strains exhibited any reduction in lag phase duration or doubling time in response to treatment with the *Pleurotus ostreatus* glucan extract compared to the control (Figure 5a,b). Similarly, *S. enterica* serovar Typhimurium showed no observable change in lag phase duration, but a slight increase in maximum optical density was noted in the extract-treated samples (Figure 5d).

In the case of *L. monocytogenes*, lag phase duration remained unchanged (t = 9.52 h in the control vs. t = 9.97 h with extract). However, extract-treated cultures reached a higher maximum OD, suggesting a potential effect on final cell density or biomass accumulation (Figure 5c). Lag phase values were as follows: *L. monocytogenes*: t =9.52 h (control) and t = 9.97 h (extract-treated) and *S. enterica* Typhimurium: t = 0.83 h (control) and t = 1.46 h (extract-treated). It is important to note that, due to non-exponential growth patterns observed in the pathogenic strains, the growth curve fitting algorithm was unable to determine precise doubling time values, and therefore, lag phase estimates may be imprecise.

These results suggest that the growth-promoting effects of fungal β-glucan extracts are selective, primarily benefiting *Lactobacillus* and *Lacticaseibacillus* strains, with minimal or no stimulation observed in potentially harmful bacteria.

## 4. Discussion

Multiple studies have demonstrated that various prebiotic compounds can stimulate the growth of probiotic bacteria, thereby enhancing growth kinetics and viability [20,21,22,23,24]. In this study, we explored whether fungal glucans, specifically those derived from *Pleurotus ostreatus* and *Lentinula edodes*, i.e., mushrooms that are widely cultivated and consumed worldwide, could elicit similar effects. Our findings indicate a clear dose-dependent stimulatory effect of mushroom polysaccharide extracts on the growth of *Lacticaseibacillus casei*, with shorter lag phases and faster doubling times observed at higher extract concentrations (Figure 1a–c). To our knowledge, this kind of dose response to fungal glucans had not been previously described in the literature, likely due to the limitations of low-throughput bacterial growth tracking methods. Studies that have observed similar effects have primarily focused on evaluating the effect of a single concentration of β-glucans and on comparing the effects on bacterial growth to those of other prebiotic substances [25,26,27]. Similarly, a recent study by Toros et al. investigated the average growth of *L. plantarum* and *L. casei* and how they were affected by freeze-dried oyster mushroom powders measured in CFU/g under different treatments [20]. There are also examples of other polysaccharides that have been shown to enhance, inhibit, or have no influence of the growth of lactobacilli. For instance, alginate, chitosan and dextran as polysaccharides have been evaluated on their prebiotic characteristics of several lactobacilli [21,22].

We observed that the sample containing the highest *Lentinula* extract concentration (1.0% *w*/*w* extract) had the shortest lag phase, while lower concentrations had longer lag phase durations (Figure 1d,e). Interestingly, although the duration of the lag phase increased with dilution, the doubling times for all samples containing mushroom glucans were relatively similar to each other and approximately half that of the samples treated with a control. This implies that higher extract concentrations cause earlier entry into the exponential growth phase; however, once the bacterial cells treated with fungal extracts enter this phase, they display similar growth kinetics. Other studies have found that specific prebiotics can reduce the doubling time of certain *Lactobacillus* and *Lacticaseibacillus* species; however, their results show more clearly dose-dependent effect, suggesting that the fungal glucans used in our research may have a different mechanism of action [23,24].

Since all of these experiments were conducted using *L. casei* as the model organism for probiotic bacteria, we extended our investigation to determine whether the stimulation effect was strain-specific or applicable to other probiotic bacterial strains. We conducted experiments with six different bacterial strains (*L. casei*, *L. helveticus*, *L. rhamnosus GG*, *L. plantarum*, *L. paracasei*, *L. paracasei* sp. *paracasei*, and *L. acidophilus*) using only the most effective 1.0% extract solution. We found that the growth-stimulating effect of fungal glucans is universal among all the strains tested. However, the magnitude varied significantly (Figure 2). Specifically, all of the species tested exhibited a significant reduction in lag phase duration. The lag phase in extract-treated samples was comparable between the strains, whereas the control sample showed more variation. On the other hand, the reduction in doubling time observed in *L. casei* was not equally present among other strains: *L. helveticus*, *L. rhamnosus GG*, and *L. acidophilus* also showed a decrease in doubling time, while *L. paracasei* and *L. paracasei* sp. *paracasei* exhibited an increase in the doubling time in the extract-treated samples compared to the control. Considering all the information obtained, we conclude that the addition of fungal glucan extracts has a strong, dose-responsive effect on growth stimulation. This effect is common to the two mushroom species used in this study—*Pleurotus ostreatus* and *Lentinula edodes*, both of which are rich in β-glucans—and applies to all probiotic strains tested, albeit to varying degrees.

To investigate the impact of different structural types of fungal β-glucans on the growth of probiotic bacterial strains, we conducted an experiment in which we cultured them with the addition of commercial *Saccharomyces cerevisiae* β-glucans extracts. Since *Saccharomyces* β-glucans are predominantly linear molecules with a very low degree of branching [25], this was an excellent opportunity to assess whether the type and degree of branching could be the factor determining the magnitude and mechanism of bacterial growth stimulation. *Saccharomyces* β-glucan extracts are not completely water-soluble; while β-glucan molecules are hydrophilic, they tend to aggregate in aqueous solutions. The size and molecular weight of the large glucan aggregates cause them to precipitate over time [19]. Therefore, we had to use a lower concentration of extracts (0.025% vs. 1.0%) to avoid artefacts in optical density measurements. Despite having a 40-times lower extract concentration than the 1.0% *Pleurotus* water extract, the results show a dramatic difference between the *Saccharomyces* extract-supplemented samples and the control samples (Figure 3). Not only did the extract-supplemented samples exhibit a significantly shorter lag phase, but they also grew in a manner that was practically identical to each other in terms of growth kinetics. The control samples had a much larger standard deviation of the growth curves. The extract-supplemented samples showed growth kinetics changes comparable to those observed with *Pleurotus* extracts: a shortening of the lag phase by 7 to 8 h (in all samples) and a reduction in doubling time in *L. casei* and *L. rhamnosus GG* samples. *L. paracasei* did not display an observable decrease in doubling time. This led us to the conclusion that the stimulation mechanism was comparable between *Pleurotus* extracts and *Saccharomyces* extracts, that is that the same type of stimulation occurs with both extracts, regardless of their significantly different β-glucan compositions. This finding was exciting since, although *Saccharomyces* β-glucans are regarded as potential prebiotic compounds [26], there are almost no studies that show prebiotic effects of *Saccharomyces* β-glucans as strong as those observed in this research but rather only weak prebiotic effects not strong enough to classify *Saccharomyces* β-glucans as novel potential prebiotics [27].

The final confirmation that it was the β-glucan fraction of the yeast extract that stimulated growth came from an experiment comparing bacterial growth kinetics with and without supplementation with a *Saccharomyces* β-glucan standard preparation, its supernatant (collected after overnight precipitation of insoluble particles), and an autolyzed water-soluble *Saccharomyces* extract (Figure 4). Both the *Saccharomyces* β-glucan standard preparation and its supernatant showed a growth kinetics stimulation profile comparable to our results with extracts (with a much larger standard deviation in the whole extract samples compared to only the supernatant samples, which could be attributed to a much less homogenous solution of the initial standard preparation compared to its supernatant, containing both water-soluble and insoluble fractions as well as different sizes and shapes of aggregates). The autolyzed *Saccharomyces* extract did not stimulate bacterial growth in the same manner; however, it caused a decrease in growth rates. The autolyzed extract contains the total water-soluble content of yeast cells (vitamins, amino acids, peptides, nucleosides, and carbohydrates), which means that it also includes β-glucans; however, they are in their natural form, i.e., complexed with proteins, which makes them less available and prevents them from aggregating and producing larger β-glucan particles. The β-glucan standard preparation contains only purified β-glucans and other yeast carbohydrates. Taking this into consideration, we concluded that the stimulation effect could only be attributed to the β-glucan fraction of the yeast cells and that only highly purified and concentrated β-glucans are capable of producing this effect.

Several enzymes are involved in the metabolism of β-glucans in *lactic acid* bacteria. Notably, β-glucanases, including endo-β-1,3- and endo-β-1,4-glucanases, initiate the breakdown of β-glucan polymers by cleaving internal glycosidic bonds [28,29]. Subsequently, β-glucosidase catalyzes the hydrolysis of β-glucosidic linkages in smaller oligosaccharides, releasing glucose units. Genomic studies have identified genes such as *bglA*, *bglB*, and *glvA* to be associated with these enzymatic activities in lactic acid bacteria, supporting the idea that β-glucans can be utilized as fermentable substrates in some *Lactobacillus* [30]. These findings suggest that the stimulatory effect observed in our study may be mediated, at least in part, by such enzymatic mechanisms.

Given our aim to develop new functional foods and food supplements, it is crucial to evaluate the impact of these new prebiotic compounds on pathogenic bacteria and opportunistic pathogens present in the human gastrointestinal tract, as the stimulation of their growth could pose severe health risks. There is limited information in the literature on the effect of fungal glucans and similar compounds on pathogenic bacteria. A study by Shi and collaborators showed that *E. coli* did not grow when provided with only glucooligosaccharides or inulin as the sole carbon source, indicating the bacterium did not metabolize them [31]. Furthermore, certain oligosaccharides have been shown to inhibit the adhesion of *E. coli* to human gastrointestinal tract cells [32,33,34]. We tested the effect of *Pleurotus* extract on the growth of three pathogenic/opportunistic bacterial species: *E. coli*, *L. monocytogenes*, and *S. enterica* Typhimurium. There was no difference between the growth kinetics of the two different *E. coli* strains, as indicated by lag phase duration, doubling time, or final optical density (Figure 5). Thus, *Pleurotus ostreatus* extract does not affect the growth of *E. coli* and poses no health hazard. *S. enterica* Typhimurium and *L. monocytogenes* did not exhibit any noticeable reduction in lag phase duration or doubling time, although changes in the final optical density were observed (Figure 5). *S. enterica* Typhimurium had a slightly increased final optical density compared to the control, while *L. monocytogenes* had a considerable increase. Since the β-glucans did not stimulate faster growth of the samples, the increase in the final optical density (i.e., the final concentration of bacterial cells) is likely a consequence of additional nutrients in the growth medium. This suggests that *S. enterica* Typhimurium and *L. monocytogenes* can break down β-glucans using specific glucanase enzymes, utilizing the resulting glucose as an energy source. This is consistent with the results of Ebersbach and collaborators, who found that certain *L. monocytogenes* strains can ferment specific polysaccharide prebiotic compounds [35]. However, the study authors noted no correlation between the ability to ferment carbohydrates in vitro and infectivity in animal models, suggesting that interactions between pathogenic bacteria and different carbohydrate polymers may be strain-specific. Additionally, *L. monocytogenes*, a Gram-positive bacterium, may utilize fungal glucans as a food source or for cell wall construction, unlike the Gram-negative *E. coli* and *S. enterica Typhimurium*, which have smaller cell walls composed chiefly of polysaccharides, including β-glucans [36].

In conclusion, fungal polysaccharide extracts, in particular fungal β-glucans, can stimulate the growth and proliferation of specific probiotic bacterial strains, leading to shorter lag phases and faster doubling times. The effect of growth stimulation is strain-specific, meaning that different strains have different affinities towards structurally distinct types of fungal glucans. In addition, this study presents a straightforward and efficient method for the rapid screening of optimal strain–glucan combinations, facilitating the development of innovative synbiotic formulations.

## Data Availability

The original contributions presented in this study are included in the article. Further inquiries can be directed to the corresponding author.
